# Supplementary material for: The Warps and Wefts of a Polyploidy Complex: Integrative Species Delimitation of the Diploid Leucanthemum (Compositae, Anthemideae) Representatives
Source: Plants (Basel). 2022 Jul 19;11(14):1878. doi: 10.3390/plants11141878 (PMC9319895; doi:10.3390/plants11141878)
Supplement: Supplementary file 1 [file plants-11-01878-s001.zip › supplementary/Figure_S3.pdf]

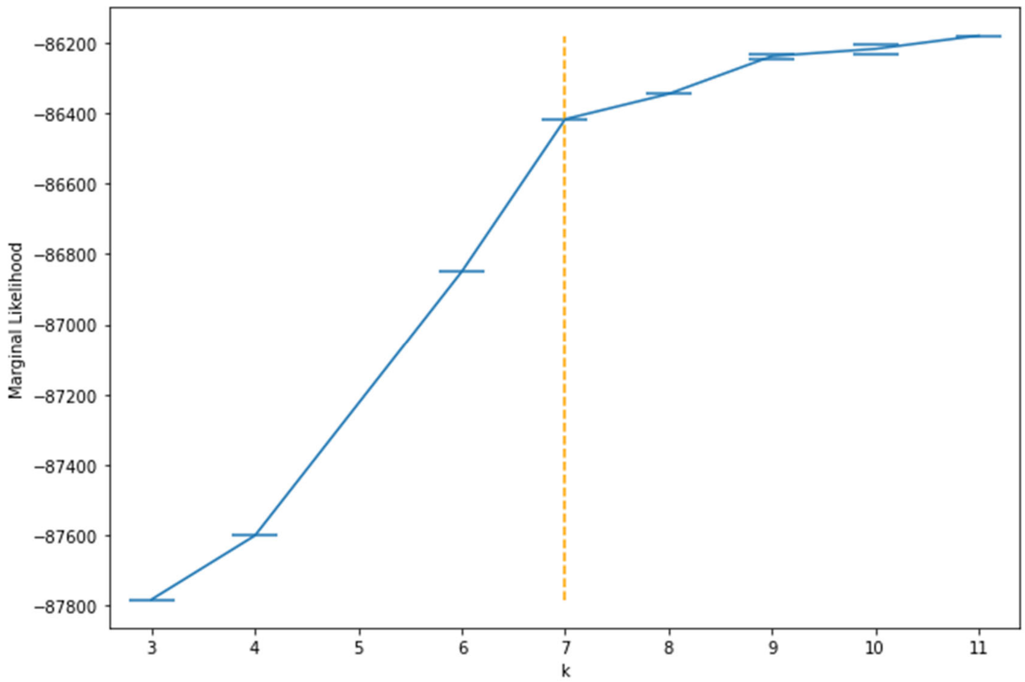

Figure S3. Marginal likelihood (ML) of the SNAPP species scenarios plotted against respective model complexities. Model complexity is the number of species in the scenario. The orange dashed line indicates the knee point in the ML-complexity curve.
