# Supplementary figures and images for: The Warps and Wefts of a Polyploidy Complex: Integrative Species Delimitation of the Diploid Leucanthemum (Compositae, Anthemideae) Representatives
Source: Plants (Basel). 2022 Jul 19;11(14):1878. doi: 10.3390/plants11141878 (PMC9319895; doi:10.3390/plants11141878)

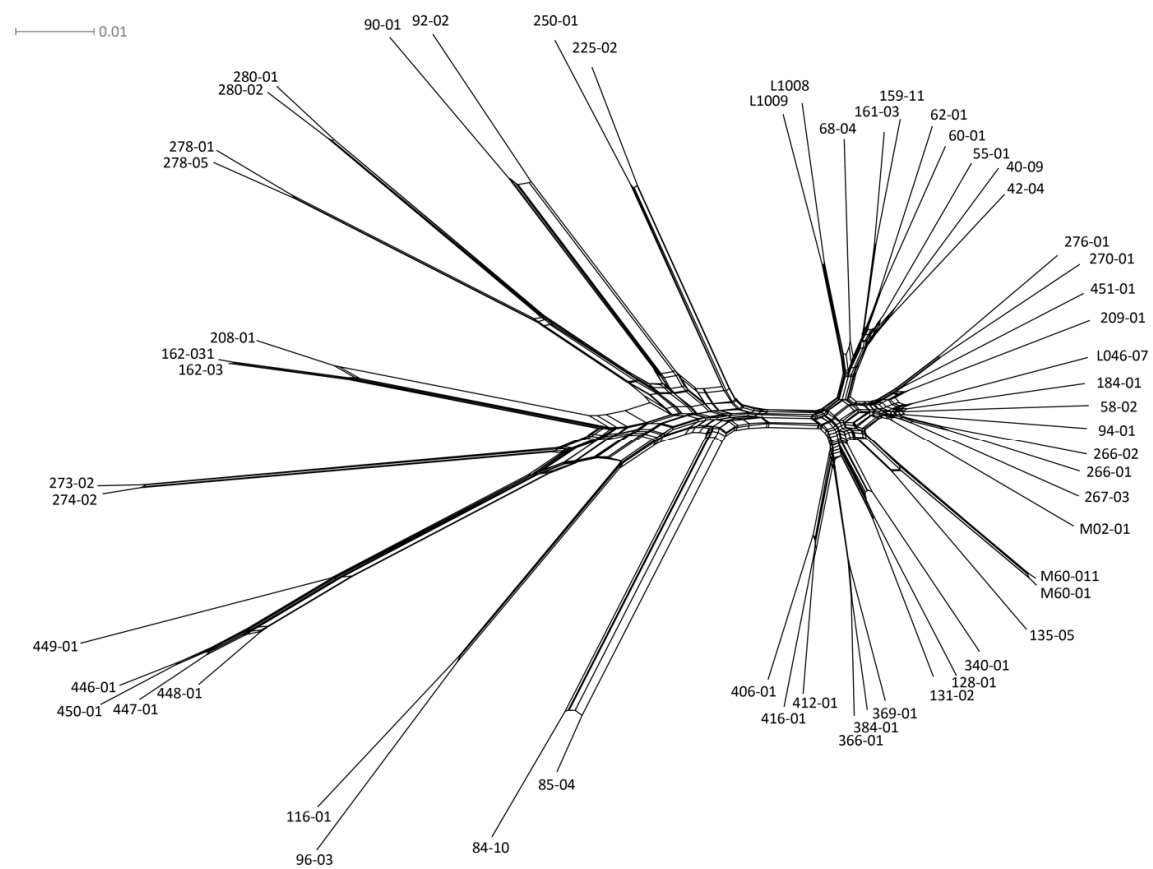

**Figure S2.** NeighborNet network based on nucleotide Nei distances.

Supplement: Supplementary file 1 [file plants-11-01878-s001.zip › supplementary/Figure_S2.pdf]
